# *catena*-Poly[[[(1,10-phenanthroline)copper(I)]-μ-cyanido] ethanol hemisolvate]

**DOI:** 10.1107/S1600536810045186

**Published:** 2010-11-10

**Authors:** Jianhua Nie, Jun Wang, Chuntao Dai

**Affiliations:** a Zhongshan Polytechnic, Zhongshan, Guangdong 528404, People’s Republic of China

## Abstract

In the title coordination polymer, {[Cu(CN)(C_12_H_10_N_2_)]·0.5C_2_H_5_OH}_*n*_, there are two Cu^I^ ions, two 1,10-phenanthroline (phen) ligands and two cyanide ions in the asymmetric unit along with a highly disordered ethanol solvent mol­ecule, which was modelled as being disordered over two sets of sites in a 0.829 (7):0.171 (7) ratio. The orientation/ordering of the C and N atoms of the cyanide ions could not be determined in the present refinement and they were modelled as being statistically disordered. Both copper ions are coordinated by an *N*,*N*′-bidentate phen ligand and two cyanide ligands, generating distorted tetra­hedral CuN_2_ *Q*_2_ (*Q* = C or N) tetra­hedra. The μ-cyanide ligands link the metal ions, forming a zigzag chain propagating in [001]. The chains are cross-linked by numerous aromatic π–π stacking contacts between adjacent phen rings [minimum centroid–centroid separation = 3.620 (3) Å].

## Related literature

For general background to cyanide coordination polymers, see: Holmes & Girolami (1999 ▶); Deng *et al.* (2008 ▶). For related structures, see: Dyason *et al.* (1985 ▶); Chesnut *et al.* (1999 ▶); Zhao *et al.* (2004 ▶); Huang *et al.* (2004 ▶).
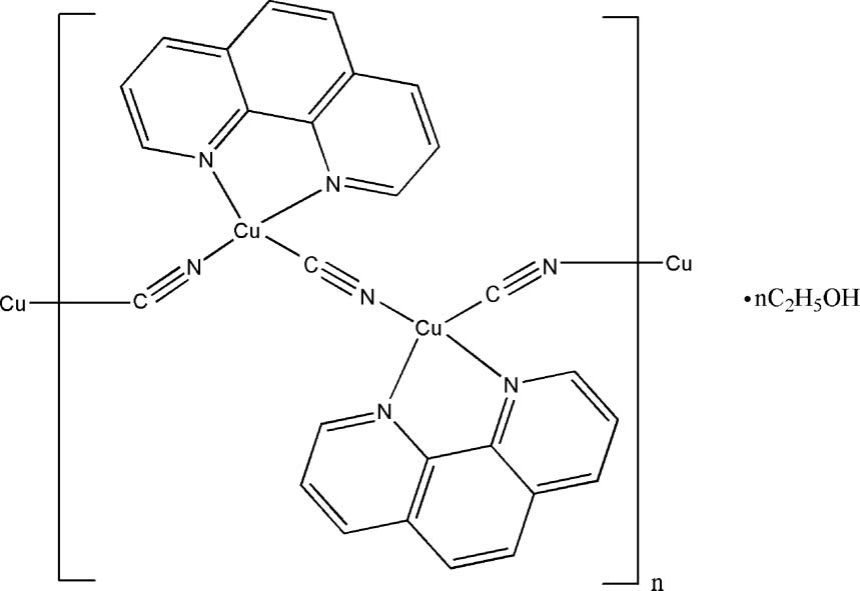


## Experimental

### 

#### Crystal data

[Cu(CN)(C_12_H_10_N_2_)]·0.5C_2_H_6_O
*M* *_r_* = 292.8
Monoclinic, 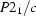

*a* = 18.4896 (6) Å
*b* = 8.4033 (3) Å
*c* = 16.5166 (5) Å
β = 109.974 (2)°
*V* = 2411.88 (14) Å^3^
*Z* = 8
Mo *K*α radiation
μ = 1.80 mm^−1^
*T* = 293 K
0.25 × 0.23 × 0.19 mm

#### Data collection

Bruker APEXII area-detector diffractometer
Absorption correction: multi-scan (*SADABS*; Bruker, 2004 ▶) *T*_min_ = 0.662, *T*_max_ = 0.726
21068 measured reflections
4729 independent reflections
2624 reflections with *I* > 2σ(*I*)
*R*_int_ = 0.090

#### Refinement

*R*[*F* ^2^ > 2σ(*F* ^2^)] = 0.057
*wR*(*F* ^2^) = 0.159
*S* = 1.02
4729 reflections
344 parameters
65 restraints
H-atom parameters constrained
Δρ_max_ = 0.37 e Å^−3^
Δρ_min_ = −0.53 e Å^−3^

### 

Data collection: *APEX2* (Bruker, 2004 ▶); cell refinement: *SAINT* (Bruker, 2004 ▶); data reduction: *SAINT*; program(s) used to solve structure: *SHELXS97* (Sheldrick, 2008 ▶); program(s) used to refine structure: *SHELXL97* (Sheldrick, 2008 ▶); molecular graphics: *SHELXTL* (Sheldrick, 2008 ▶); software used to prepare material for publication: *SHELXTL*.

## Supplementary Material

Crystal structure: contains datablocks I, global. DOI: 10.1107/S1600536810045186/hb5723sup1.cif

Structure factors: contains datablocks I. DOI: 10.1107/S1600536810045186/hb5723Isup2.hkl

Additional supplementary materials:  crystallographic information; 3D view; checkCIF report

## Figures and Tables

**Table 1:** Selected bond lengths (Å)

Cu1—C1/N1	1.910 (6)
Cu1—C2′/N2′	1.944 (6)
Cu1—N4	2.108 (5)
Cu1—N3	2.142 (4)
Cu2—C2^i^/N2^i^	1.909 (6)
Cu2—C1′/N1′	1.921 (5)
Cu2—N6	2.126 (5)
Cu2—N5	2.130 (4)

Symmetry code: (i) 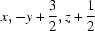.

**Fig. 1.**
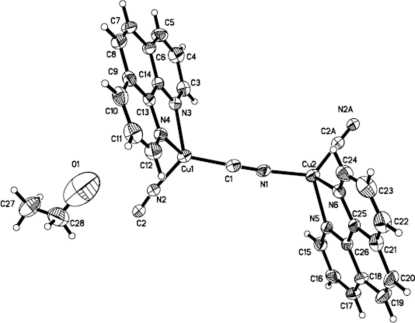
ORTEP represention of (I), showing 30% probability displacement ellipsoids. Symmetry codes: (a) x, 1.5-y, 0.5+z.

**Fig. 2.**
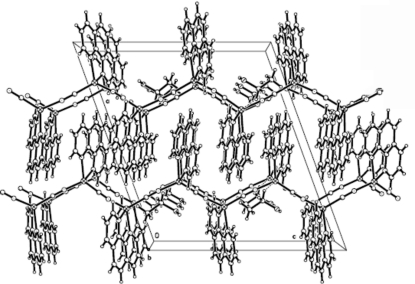
View of the three-dimensional structure of the title compound.

## supplementary crystallographic information

### Comment

Metal coordination polymer based on cyanide group have raised intense interest due to their structural diversity and their potential applications in magnetic materials (Holmes & Girolami, 1999; Deng *et al.*, 2008). Up to date, a large number of one-, two-, and three-dimensional coordination polymers have been prepared by the choice of metal-cyanide bridging centers and versatile secondary ligands such as 1,10-phenanthroline and 2,2-pyridine (Dyason *et al.*, 1985; Chesnut *et al.*, 1999; Zhao *et al.*, 2004; Huang *et al.*, 2004). Herein, we obtained one copper coordination polymer of [Cu(C_12_H_10_N_2_)(CN).C_2_H_6_O]_n_ under hydrothermal condition, and its structure was reported.

As depicted in Fig. 1, each Cu^i^ ion is four-coordianted by two N atoms from one 1,10-phenanthroline (phen) ligand and two cyano ligands. The Cu-phen subunits are in turn interconnected by /m_2_-cyano ligands to form a 1D zigzag chain. These chains are further assembled by /p···/p stacking contacts between adjacent phen rings and extend to form a three-dimensional supramolecular network (Fig. 2). The interplanar distance between them is ca. 3.60 Å (symmetry operator for the 1,10-phenanthroline ligand: 1-*x*, 1-*y*, 2-*z*). The lattice ethanol molecule is independently disordered over two parts of 0.829 (7): 0.171 (7). (see refinement section for details).

### Experimental

Copper(I) cyanide (0.089 g, 1 mmol) and 1,10-phenanthroline (0.1801 g, 1 mmol) were added to a mixture of water (5 ml) and ethanol (5 ml). The resultant mixture was sealed in a 20 ml stainless steel reactor with a Teflon liner and kept under autogenous pressure at 413 K for 48 h, and then cooled to room temperature at a rate of 5 K/min. Yellow blocks of (I) formed with a yield of approximately 58% based on 1,10-phenanthroline.

### Refinement

The lattice ethanol molecules are arranged as symmetry related pairs around a center of inversion. In the original refinement the molecule showed significantly elongated thermal ellipsoids indicating disorder. The ethanol molecule was thus refined as being disordered over two sites in a ratio of 0.829 (7): 0.171 (7). Due to the significant overlap of the disordered atoms the following restraints and constraints were applied: The adps of the disordered atoms were restrained to be close to isotropic and those of equivalent atoms were set to be identical.

The C and N atoms of each bridging cyano groups are ambiguous and were refined to be same ratios and their equivalent atoms were set to be identical.

All H atoms attached to C atoms were fixed geometrically and treated as riding with C—H = 0.93-0.97 and O—H = 1.2 Å, and U_iso_(H) = 1.2U_eq_(C) and U_iso_(H) = 1.5U_eq_(O).

### Crystal data

**Table:**

[Cu(CN)(C_12_H_10_N_2_)]·0.5C_2_H_6_O	*F*(000) = 1192
*M**_r_* = 292.8	*D*_x_ = 1.613 Mg m^−^^3^
Monoclinic, *P*2_1_/*c*	Mo *K*α radiation, λ = 0.71073 Å
Hall symbol: -P 2ybc	Cell parameters from 4800 reflections
*a* = 18.4896 (6) Å	θ = 1.4–28.0°
*b* = 8.4033 (3) Å	µ = 1.80 mm^−^^1^
*c* = 16.5166 (5) Å	*T* = 293 K
β = 109.974 (2)°	Block, yellow
*V* = 2411.88 (14) Å^3^	0.25 × 0.23 × 0.19 mm
*Z* = 8	

### Data collection

**Table:**

Bruker APEXII area-detector diffractometer	4729 independent reflections
Radiation source: fine-focus sealed tube	2624 reflections with *I* > 2σ(*I*)
graphite	*R*_int_ = 0.090
φ and ω scans	θ_max_ = 26.0°, θ_min_ = 2.3°
Absorption correction: multi-scan (*SADABS*; Bruker, 2004)	*h* = −22→22
*T*_min_ = 0.662, *T*_max_ = 0.726	*k* = −9→10
21068 measured reflections	*l* = −20→20

### Refinement

**Table:**

Refinement on *F*^2^	Primary atom site location: structure-invariant direct methods
Least-squares matrix: full	Secondary atom site location: difference Fourier map
*R*[*F*^2^ > 2σ(*F*^2^)] = 0.057	Hydrogen site location: inferred from neighbouring sites
*wR*(*F*^2^) = 0.159	H-atom parameters constrained
*S* = 1.02	*w* = 1/[σ^2^(*F*_o_^2^) + (0.0686*P*)^2^ + 1.9113*P*] where *P* = (*F*_o_^2^ + 2*F*_c_^2^)/3
4729 reflections	(Δ/σ)_max_ = 0.004
344 parameters	Δρ_max_ = 0.37 e Å^−^^3^
65 restraints	Δρ_min_ = −0.53 e Å^−^^3^

### Special details

**Table:**

Geometry. All esds (except the esd in the dihedral angle between two l.s. planes) are estimated using the full covariance matrix. The cell esds are taken into account individually in the estimation of esds in distances, angles and torsion angles; correlations between esds in cell parameters are only used when they are defined by crystal symmetry. An approximate (isotropic) treatment of cell esds is used for estimating esds involving l.s. planes.
Refinement. Refinement of F^2^ against ALL reflections. The weighted R-factor wR and goodness of fit S are based on F^2^, conventional R-factors R are based on F, with F set to zero for negative F^2^. The threshold expression of F^2^ > 2sigma(F^2^) is used only for calculating R-factors(gt) etc. and is not relevant to the choice of reflections for refinement. R-factors based on F^2^ are statistically about twice as large as those based on F, and R- factors based on ALL data will be even larger.

### Fractional atomic coordinates and isotropic or equivalent isotropic displacement parameters (Å^2^)

**Table:**

	*x*	*y*	*z*	*U*_iso_*/*U*_eq_	Occ. (<1)
Cu1	0.19744 (4)	0.57901 (10)	0.46043 (5)	0.0533 (3)	
Cu2	0.32714 (4)	0.65348 (10)	0.77685 (4)	0.0523 (3)	
C1	0.2519 (3)	0.5967 (7)	0.5813 (4)	0.0557 (15)	0.50
N1'	0.2519 (3)	0.5967 (7)	0.5813 (4)	0.0557 (15)	0.50
C2	0.2732 (3)	0.7392 (7)	0.3409 (3)	0.0470 (13)	0.50
N2'	0.2732 (3)	0.7392 (7)	0.3409 (3)	0.0470 (13)	0.50
N1	0.2828 (3)	0.6179 (7)	0.6549 (3)	0.0501 (14)	0.50
C1'	0.2828 (3)	0.6179 (7)	0.6549 (3)	0.0501 (14)	0.50
N2	0.2420 (3)	0.6744 (7)	0.3810 (3)	0.0477 (14)	0.50
C2'	0.2420 (3)	0.6744 (7)	0.3810 (3)	0.0477 (14)	0.50
C3	0.0414 (4)	0.7502 (8)	0.4464 (4)	0.0511 (16)	
H3	0.0709	0.8383	0.4719	0.061*	
C4	−0.0382 (4)	0.7600 (9)	0.4252 (4)	0.0624 (18)	
H4	−0.0610	0.8524	0.4363	0.075*	
C5	−0.0817 (4)	0.6332 (10)	0.3883 (4)	0.0644 (19)	
H5	−0.1349	0.6380	0.3736	0.077*	
C6	−0.0470 (3)	0.4939 (8)	0.3722 (3)	0.0472 (15)	
C7	−0.0889 (4)	0.3551 (10)	0.3317 (4)	0.0605 (19)	
H7	−0.1423	0.3555	0.3143	0.073*	
C8	−0.0529 (4)	0.2249 (9)	0.3185 (4)	0.0594 (18)	
H8	−0.0818	0.1375	0.2911	0.071*	
C9	0.0284 (4)	0.2175 (8)	0.3455 (3)	0.0491 (15)	
C10	0.0687 (4)	0.0835 (9)	0.3361 (4)	0.0640 (19)	
H10	0.0424	−0.0067	0.3086	0.077*	
C11	0.1467 (5)	0.0854 (9)	0.3675 (5)	0.071 (2)	
H11	0.1741	−0.0056	0.3639	0.085*	
C12	0.1861 (4)	0.2228 (9)	0.4050 (4)	0.0619 (18)	
H12	0.2396	0.2219	0.4249	0.074*	
C13	0.0718 (3)	0.3522 (7)	0.3842 (3)	0.0390 (13)	
C14	0.0331 (3)	0.4941 (7)	0.3959 (3)	0.0397 (13)	
C15	0.4760 (4)	0.8556 (8)	0.8029 (4)	0.0519 (16)	
H15	0.4437	0.9339	0.7698	0.062*	
C16	0.5546 (4)	0.8874 (9)	0.8372 (4)	0.0601 (18)	
H16	0.5737	0.9853	0.8276	0.072*	
C17	0.6029 (3)	0.7741 (9)	0.8847 (4)	0.0586 (18)	
H17	0.6554	0.7944	0.9081	0.070*	
C18	0.5739 (3)	0.6280 (8)	0.8983 (4)	0.0513 (17)	
C19	0.6205 (4)	0.5004 (10)	0.9452 (4)	0.0663 (19)	
H19	0.6735	0.5141	0.9693	0.080*	
C20	0.5893 (4)	0.3616 (10)	0.9550 (4)	0.070 (2)	
H20	0.6211	0.2808	0.9862	0.084*	
C21	0.5079 (4)	0.3337 (8)	0.9189 (4)	0.0519 (16)	
C22	0.4723 (5)	0.1913 (10)	0.9253 (5)	0.074 (2)	
H22	0.5018	0.1072	0.9559	0.089*	
C23	0.3964 (5)	0.1732 (9)	0.8881 (5)	0.078 (2)	
H23	0.3729	0.0772	0.8924	0.094*	
C24	0.3532 (4)	0.2993 (9)	0.8432 (4)	0.0617 (18)	
H24	0.3005	0.2848	0.8168	0.074*	
C25	0.4607 (3)	0.4565 (7)	0.8732 (3)	0.0419 (14)	
C26	0.4937 (3)	0.6066 (7)	0.8619 (3)	0.0390 (14)	
N3	0.0772 (2)	0.6226 (6)	0.4324 (3)	0.0404 (12)	
N4	0.1499 (3)	0.3554 (6)	0.4134 (3)	0.0466 (12)	
N5	0.4451 (2)	0.7205 (6)	0.8149 (3)	0.0392 (11)	
N6	0.3829 (3)	0.4406 (6)	0.8355 (3)	0.0424 (12)	
C27	0.1951 (6)	0.5287 (13)	0.0858 (7)	0.100 (4)	0.829 (7)
H27A	0.2274	0.5476	0.0520	0.150*	0.829 (7)
H27B	0.1758	0.6283	0.0984	0.150*	0.829 (7)
H27C	0.1528	0.4620	0.0541	0.150*	0.829 (7)
C28	0.2396 (8)	0.450 (4)	0.1658 (10)	0.112 (6)	0.829 (7)
H28A	0.2562	0.3474	0.1522	0.135*	0.829 (7)
H28B	0.2853	0.5131	0.1940	0.135*	0.829 (7)
O1	0.2007 (8)	0.4284 (18)	0.2226 (10)	0.274 (9)	0.829 (7)
H1	0.1873	0.5150	0.2355	0.411*	0.829 (7)
C27'	0.230 (3)	0.475 (17)	0.147 (6)	0.100 (4)	0.171 (7)
H27D	0.2071	0.3846	0.1122	0.150*	0.171 (7)
H27E	0.1988	0.5674	0.1261	0.150*	0.171 (7)
H27F	0.2345	0.4553	0.2057	0.150*	0.171 (7)
C28'	0.309 (3)	0.503 (8)	0.142 (3)	0.112 (6)	0.171 (7)
H28C	0.3146	0.4410	0.0951	0.135*	0.171 (7)
H28D	0.3145	0.6143	0.1304	0.135*	0.171 (7)
O1'	0.367 (3)	0.459 (8)	0.220 (4)	0.274 (9)	0.171 (7)
H1'	0.3630	0.3649	0.2298	0.411*	0.171 (7)

### Atomic displacement parameters (Å^2^)

**Table:**

	*U*^11^	*U*^22^	*U*^33^	*U*^12^	*U*^13^	*U*^23^
Cu1	0.0405 (4)	0.0661 (6)	0.0510 (4)	−0.0090 (4)	0.0126 (3)	−0.0044 (4)
Cu2	0.0375 (4)	0.0655 (6)	0.0503 (4)	0.0059 (4)	0.0103 (3)	−0.0013 (4)
C1	0.038 (3)	0.074 (4)	0.053 (3)	0.005 (3)	0.013 (3)	0.002 (3)
N1'	0.038 (3)	0.074 (4)	0.053 (3)	0.005 (3)	0.013 (3)	0.002 (3)
C2	0.039 (3)	0.054 (4)	0.044 (3)	−0.001 (3)	0.009 (2)	−0.001 (3)
N2'	0.039 (3)	0.054 (4)	0.044 (3)	−0.001 (3)	0.009 (2)	−0.001 (3)
C3	0.064 (4)	0.041 (4)	0.049 (3)	0.002 (3)	0.020 (3)	0.003 (3)
C4	0.063 (5)	0.054 (5)	0.071 (4)	0.021 (4)	0.024 (4)	0.008 (4)
C5	0.042 (4)	0.082 (6)	0.069 (4)	0.012 (4)	0.019 (3)	0.014 (4)
C6	0.037 (3)	0.059 (4)	0.044 (3)	−0.003 (3)	0.012 (2)	0.009 (3)
C7	0.036 (3)	0.086 (6)	0.055 (4)	−0.017 (4)	0.011 (3)	0.008 (4)
C8	0.056 (4)	0.063 (5)	0.057 (4)	−0.020 (4)	0.016 (3)	−0.008 (4)
C9	0.061 (4)	0.047 (4)	0.045 (3)	−0.009 (3)	0.025 (3)	−0.004 (3)
C10	0.086 (6)	0.056 (5)	0.056 (4)	−0.009 (4)	0.031 (4)	−0.008 (3)
C11	0.094 (6)	0.050 (5)	0.076 (5)	0.017 (4)	0.038 (4)	−0.001 (4)
C12	0.062 (4)	0.061 (5)	0.063 (4)	0.018 (4)	0.022 (3)	0.003 (4)
C13	0.042 (3)	0.043 (4)	0.035 (3)	−0.001 (3)	0.017 (2)	0.001 (3)
C14	0.038 (3)	0.046 (4)	0.035 (3)	−0.001 (3)	0.012 (2)	0.003 (3)
C15	0.055 (4)	0.053 (4)	0.049 (3)	−0.007 (3)	0.019 (3)	0.004 (3)
C16	0.059 (4)	0.067 (5)	0.064 (4)	−0.022 (4)	0.033 (3)	−0.001 (4)
C17	0.036 (3)	0.080 (5)	0.060 (4)	−0.017 (4)	0.015 (3)	−0.008 (4)
C18	0.032 (3)	0.072 (5)	0.048 (3)	0.001 (3)	0.011 (3)	−0.005 (3)
C19	0.039 (4)	0.084 (6)	0.061 (4)	0.010 (4)	−0.002 (3)	0.003 (4)
C20	0.060 (5)	0.087 (6)	0.052 (4)	0.034 (4)	0.004 (3)	0.011 (4)
C21	0.069 (4)	0.041 (4)	0.046 (3)	0.014 (3)	0.019 (3)	0.003 (3)
C22	0.097 (6)	0.059 (6)	0.068 (4)	0.021 (5)	0.031 (4)	0.009 (4)
C23	0.110 (7)	0.045 (5)	0.094 (6)	−0.010 (5)	0.053 (5)	0.009 (4)
C24	0.065 (4)	0.061 (5)	0.064 (4)	−0.021 (4)	0.029 (3)	−0.010 (4)
C25	0.041 (3)	0.052 (4)	0.032 (3)	0.005 (3)	0.011 (2)	−0.006 (3)
C26	0.036 (3)	0.048 (4)	0.033 (3)	−0.002 (3)	0.011 (2)	−0.008 (3)
N1	0.033 (3)	0.064 (4)	0.052 (3)	0.010 (3)	0.013 (2)	−0.009 (3)
C1'	0.033 (3)	0.064 (4)	0.052 (3)	0.010 (3)	0.013 (2)	−0.009 (3)
N2	0.036 (3)	0.060 (4)	0.044 (3)	−0.003 (3)	0.011 (2)	−0.001 (3)
C2'	0.036 (3)	0.060 (4)	0.044 (3)	−0.003 (3)	0.011 (2)	−0.001 (3)
N3	0.041 (3)	0.041 (3)	0.038 (2)	0.000 (2)	0.013 (2)	0.000 (2)
N4	0.040 (3)	0.058 (4)	0.042 (2)	0.010 (3)	0.015 (2)	−0.002 (2)
N5	0.039 (3)	0.041 (3)	0.036 (2)	0.003 (2)	0.011 (2)	0.000 (2)
N6	0.047 (3)	0.044 (3)	0.041 (2)	−0.006 (2)	0.020 (2)	−0.004 (2)
C27	0.084 (8)	0.078 (8)	0.118 (9)	−0.019 (6)	0.008 (6)	−0.006 (7)
C28	0.100 (10)	0.134 (17)	0.119 (13)	−0.017 (9)	0.058 (9)	−0.018 (11)
O1	0.205 (14)	0.190 (14)	0.35 (2)	0.042 (11)	0.002 (13)	−0.103 (14)
C27'	0.084 (8)	0.078 (8)	0.118 (9)	−0.019 (6)	0.008 (6)	−0.006 (7)
C28'	0.100 (10)	0.134 (17)	0.119 (13)	−0.017 (9)	0.058 (9)	−0.018 (11)
O1'	0.205 (14)	0.190 (14)	0.35 (2)	0.042 (11)	0.002 (13)	−0.103 (14)

### Geometric parameters (Å, °)

**Table:**

Cu1—C1	1.910 (6)	C15—C16	1.393 (8)
Cu1—N1'	1.910 (6)	C15—H15	0.9300
Cu1—N2	1.944 (6)	C16—C17	1.357 (9)
Cu1—C2'	1.944 (6)	C16—H16	0.9300
Cu1—N4	2.108 (5)	C17—C18	1.389 (9)
Cu1—N3	2.142 (4)	C17—H17	0.9300
Cu2—N2'^i^	1.909 (6)	C18—C26	1.409 (7)
Cu2—C2^i^	1.909 (6)	C18—C19	1.427 (9)
Cu2—N1	1.921 (5)	C19—C20	1.336 (10)
Cu2—C1'	1.921 (5)	C19—H19	0.9300
Cu2—N6	2.126 (5)	C20—C21	1.435 (9)
Cu2—N5	2.130 (4)	C20—H20	0.9300
C1—N1	1.167 (7)	C21—C22	1.387 (10)
C2—N2	1.154 (6)	C21—C25	1.395 (8)
C2—Cu2^ii^	1.909 (6)	C22—C23	1.335 (10)
C3—N3	1.322 (7)	C22—H22	0.9300
C3—C4	1.395 (9)	C23—C24	1.380 (10)
C3—H3	0.9300	C23—H23	0.9300
C4—C5	1.348 (9)	C24—N6	1.332 (8)
C4—H4	0.9300	C24—H24	0.9300
C5—C6	1.405 (9)	C25—N6	1.365 (7)
C5—H5	0.9300	C25—C26	1.441 (8)
C6—C14	1.395 (7)	C26—N5	1.358 (7)
C6—C7	1.433 (9)	C27—C28	1.454 (9)
C7—C8	1.337 (9)	C27—H27A	0.9600
C7—H7	0.9300	C27—H27B	0.9600
C8—C9	1.416 (8)	C27—H27C	0.9600
C8—H8	0.9300	C28—O1	1.375 (9)
C9—C10	1.387 (9)	C28—H28A	0.9700
C9—C13	1.408 (8)	C28—H28B	0.9700
C10—C11	1.356 (9)	O1—H1	0.8200
C10—H10	0.9300	C27'—C28'	1.503 (10)
C11—C12	1.393 (10)	C27'—H27D	0.9600
C11—H11	0.9300	C27'—H27E	0.9600
C12—N4	1.331 (8)	C27'—H27F	0.9600
C12—H12	0.9300	C28'—O1'	1.415 (10)
C13—N4	1.357 (7)	C28'—H28C	0.9700
C13—C14	1.437 (8)	C28'—H28D	0.9700
C14—N3	1.363 (7)	O1'—H1'	0.8200
C15—N5	1.316 (7)		
			
C1—Cu1—N2	118.7 (2)	C20—C19—H19	119.4
C1—Cu1—N4	117.3 (2)	C18—C19—H19	119.4
N2—Cu1—N4	109.8 (2)	C19—C20—C21	121.8 (6)
C1—Cu1—N3	110.49 (19)	C19—C20—H20	119.1
N2—Cu1—N3	115.55 (19)	C21—C20—H20	119.1
N4—Cu1—N3	78.53 (18)	C22—C21—C25	116.9 (6)
C2^i^—Cu2—N1	122.6 (2)	C22—C21—C20	124.5 (7)
C2^i^—Cu2—N6	114.1 (2)	C25—C21—C20	118.6 (6)
N1—Cu2—N6	108.3 (2)	C23—C22—C21	121.0 (7)
C2^i^—Cu2—N5	112.80 (19)	C23—C22—H22	119.5
N1—Cu2—N5	112.18 (19)	C21—C22—H22	119.5
N6—Cu2—N5	78.45 (18)	C22—C23—C24	118.9 (7)
N1—C1—Cu1	175.2 (5)	C22—C23—H23	120.5
N2—C2—Cu2^ii^	178.6 (5)	C24—C23—H23	120.5
N3—C3—C4	123.5 (6)	N6—C24—C23	123.6 (7)
N3—C3—H3	118.2	N6—C24—H24	118.2
C4—C3—H3	118.2	C23—C24—H24	118.2
C5—C4—C3	118.9 (6)	N6—C25—C21	122.8 (6)
C5—C4—H4	120.6	N6—C25—C26	117.0 (5)
C3—C4—H4	120.6	C21—C25—C26	120.2 (5)
C4—C5—C6	120.2 (6)	N5—C26—C18	123.2 (6)
C4—C5—H5	119.9	N5—C26—C25	117.7 (5)
C6—C5—H5	119.9	C18—C26—C25	119.1 (5)
C14—C6—C5	117.1 (6)	C1—N1—Cu2	176.2 (5)
C14—C6—C7	119.0 (6)	C2—N2—Cu1	173.2 (5)
C5—C6—C7	123.9 (6)	C3—N3—C14	117.4 (5)
C8—C7—C6	121.4 (6)	C3—N3—Cu1	130.2 (4)
C8—C7—H7	119.3	C14—N3—Cu1	112.3 (4)
C6—C7—H7	119.3	C12—N4—C13	117.3 (6)
C7—C8—C9	121.3 (6)	C12—N4—Cu1	128.5 (4)
C7—C8—H8	119.4	C13—N4—Cu1	114.1 (4)
C9—C8—H8	119.4	C15—N5—C26	117.3 (5)
C10—C9—C13	117.4 (6)	C15—N5—Cu2	129.6 (4)
C10—C9—C8	123.6 (6)	C26—N5—Cu2	113.1 (4)
C13—C9—C8	119.1 (6)	C24—N6—C25	116.6 (5)
C11—C10—C9	119.3 (7)	C24—N6—Cu2	130.0 (4)
C11—C10—H10	120.4	C25—N6—Cu2	113.4 (4)
C9—C10—H10	120.4	C28—C27—H27A	109.5
C10—C11—C12	120.4 (7)	C28—C27—H27B	109.5
C10—C11—H11	119.8	H27A—C27—H27B	109.5
C12—C11—H11	119.8	C28—C27—H27C	109.5
N4—C12—C11	122.3 (6)	H27A—C27—H27C	109.5
N4—C12—H12	118.8	H27B—C27—H27C	109.5
C11—C12—H12	118.8	O1—C28—C27	114.6 (13)
N4—C13—C9	123.3 (6)	O1—C28—H28A	108.6
N4—C13—C14	117.0 (5)	C27—C28—H28A	108.6
C9—C13—C14	119.7 (5)	O1—C28—H28B	108.6
N3—C14—C6	122.8 (6)	C27—C28—H28B	108.6
N3—C14—C13	117.8 (5)	H28A—C28—H28B	107.6
C6—C14—C13	119.3 (5)	C28—O1—H1	109.5
N5—C15—C16	123.3 (6)	C28'—C27'—H27D	109.5
N5—C15—H15	118.3	C28'—C27'—H27E	109.5
C16—C15—H15	118.3	H27D—C27'—H27E	109.5
C17—C16—C15	119.4 (6)	C28'—C27'—H27F	109.5
C17—C16—H16	120.3	H27D—C27'—H27F	109.5
C15—C16—H16	120.3	H27E—C27'—H27F	109.5
C16—C17—C18	119.9 (6)	O1'—C28'—C27'	111.5 (16)
C16—C17—H17	120.0	O1'—C28'—H28C	109.3
C18—C17—H17	120.0	C27'—C28'—H28C	109.3
C17—C18—C26	116.9 (6)	O1'—C28'—H28D	109.3
C17—C18—C19	123.9 (6)	C27'—C28'—H28D	109.3
C26—C18—C19	119.2 (6)	H28C—C28'—H28D	108.0
C20—C19—C18	121.1 (6)	C28'—O1'—H1'	109.5

Symmetry codes: (i) *x*, −*y*+3/2, *z*+1/2; (ii) *x*, −*y*+3/2, *z*−1/2.
